# Preoperative plasma level of endoglin as a predictor for disease outcomes after radical cystectomy for nonmetastatic urothelial carcinoma of the bladder

**DOI:** 10.1002/mc.23355

**Published:** 2021-09-29

**Authors:** Ekaterina Laukhtina, Victor M. Schuettfort, David D'Andrea, Benjamin Pradere, Keiichiro Mori, Fahad Quhal, Reza Sari Motlagh, Hadi Mostafaei, Satoshi Katayama, Nico С. Grossmann, Pawel Rajwa, Flora Zeinler, Mohammad Abufaraj, Marco Moschini, Kristin Zimmermann, Pierre I. Karakiewicz, Harun Fajkovic, Douglas Scherr, Eva Compérat, Peter Nyirady, Michael Rink, Dmitry Enikeev, Shahrokh F. Shariat

**Affiliations:** ^1^ Department of Urology, Comprehensive Cancer Center Medical University of Vienna Vienna Austria; ^2^ Institute for Urology and Reproductive Health Sechenov University Moscow Russia; ^3^ Department of Urology University Medical Center Hamburg‐Eppendorf Hamburg Germany; ^4^ Department of Urology The Jikei University School of Medicine Tokyo Japan; ^5^ Department of Urology King Fahad Specialist Hospital Dammam Saudi Arabia; ^6^ Men's Health and Reproductive Health Research Center Shahid Beheshti University of Medical Sciences Tehran Iran; ^7^ Research Center for Evidence Based Medicine Tabriz University of Medical Sciences Tabriz Iran; ^8^ Department of Urology Okayama University Graduate School of Medicine, Dentistry and Pharmaceutical Sciences Okayama Japan; ^9^ Department of Urology University Hospital Zurich Zurich Switzerland; ^10^ Department of Urology Medical University of Silesia Zabrze Poland; ^11^ Department of Special Surgery, Division of Urology Jordan University Hospital, The University of Jordan Amman Jordan; ^12^ Department of Urology Luzerner Kantonsspital Lucerne Switzerland; ^13^ Department of Urology and Division of Experimental Oncology, Urological Research Institute Vita‐Salute San Raffaele; ^14^ Department of Urology Federal Armed Services Hospital Koblenz Koblenz Germany; ^15^ Division of Urology, Cancer Prognostics and Health Outcomes Unit University of Montreal Health Center Montreal Canada; ^16^ Karl Landsteiner Institute of Urology and Andrology Vienna Austria; ^17^ Department of Urology, Weill Cornell Medical College New York Presbyterian Hospital New York New York USA; ^18^ Department of Urology Semmelweis University Budapest Hungary; ^19^ Department of Urology Weill Cornell Medical College New York New York USA; ^20^ Department of Urology University of Texas Southwestern Dallas Texas USA; ^21^ Department of Urology, Second Faculty of Medicine Charles University Prague Czech Republic; ^22^ Hourani Center for Applied Scientific Research Al‐Ahliyya Amman University Amman Jordan

**Keywords:** biomarker, bladder cancer, endoglin, radical cystectomy

## Abstract

Elevated preoperative plasma level of endoglin has been associated with worse oncologic outcomes in various malignancies. The present large‐scale study aimed to determine the predictive and prognostic values of preoperative endoglin with regard to clinicopathologic and survival outcomes in patients treated with radical cystectomy (RC) for nonmetastatic urothelial carcinoma of the bladder (UCB). We prospectively collected preoperative blood samples from 1036 consecutive patients treated with RC for UCB. Logistic and Cox regression analyses were undertaken to assess the correlation of endoglin levels with pathologic and survival outcomes, respectively. The AUC and C‐index were used to assess the discrimination. Patients with adverse pathologic features had significantly higher median preoperative endoglin plasma levels than their counterparts. Higher preoperative endoglin level was independently associated with an increased risk for lymph node metastasis, ≥pT3 disease, and nonorgan confined disease (NOCD; all *p* < 0.001). Plasma endoglin level was also independently associated with cancer‐specific and overall survival in both pre‐ and postoperative models (all *p* < 0.05), as well as with recurrence‐free survival (RFS) in the preoperative model (*p* < 0.001). The addition of endoglin to the preoperative standard model improved its discrimination for prediction of lymph node metastasis, ≥pT3 disease, NOCD, and RFS (differential increases in C‐indices: 10%, 5%, 5.8%, and 4%, respectively). Preoperative plasma endoglin is associated with features of biologically and clinically aggressive UCB as well as survival outcomes. Therefore, it seems to hold the potential of identifying UCB patients who may benefit from intensified therapy in addition to RC such as extended lymphadenectomy or/and preoperative systemic therapy.

## 1 INTRODUCTION

Due to the high intertumoral heterogeneity of urothelial carcinoma, a significant percentage of patients treated with radical cystectomy (RC) for urothelial carcinoma of the bladder (UCB) still experience disease progression.^1^, ^2^, ^3^ Accurate identification of patients who could benefit from intensified perioperative systemic therapy remains an unmet clinical need.^4^ Current prognostic models are mostly based on clinicopathologic features.^5^, ^6^, ^7^, ^8^, ^9^ Nevertheless, preoperative patient selection for individualized treatment and follow‐up scheduling remains challenging as we lack clinically reliable biomarkers for outcome prediction ^6^, ^9^, ^10^, ^11^, ^12^, ^13^, ^14^, ^15^, ^16^, ^17^, ^18^ To accurately predict biologically and clinically aggressive disease as well as poor survival in UCB patients, novel biomarkers need to improve the current outcome prediction by a prognostically and clinically significant margin.^19^

Angiogenesis has been proposed as a critical event in the initiation and progression of solid malignancies.^20^ Endoglin is highly expressed by human vascular endothelial cells and has been reported as a marker of angiogenesis.^21^ Elevated preoperative plasma levels of endoglin have been associated with worse oncologic outcomes in various malignancies.^22^, ^23^, ^24^ Among urological malignancies, higher blood levels of endoglin have been found to be associated with higher preoperative serum prostate‐specific antigen, adverse pathologic features, as well as biochemical progression in prostate cancer patients.^25^, ^26^, ^27^ The association of circulating levels of endoglin with bladder cancer remains, however, uninvestigated.

We hypothesized that elevated preoperative endoglin plasma levels would be associated with features of biologically and clinically aggressive UCB as well as poor survival outcomes. To test this hypothesis, we studied the predictive and prognostic values of blood levels of endoglin in a large consecutive cohort of patients with nonmetastatic UCB treated with RC and pelvic lymphadenectomy.

## 2 MATERIALS AND METHODS

### 2.1 Data source and patient cohort

All procedures described in the present study were undertaken with the approval and oversight of the Institutional Review Board for the Protection of Human Subjects (IRB: 1011011386, 069826900). This study is a retrospective analysis of a prospectively collected consecutive cohort of patients who were treated with RC for nonmetastatic UCB at two medical centers. Sample collection took place between 2003 and 2015. The exclusion criteria were the following: patients with any concomitant secondary malignancies, including upper urinary tract carcinoma, as well as patients with missing data. The extent of lymphadenectomy and choice of urinary diversion was at the surgeon's discretion. No patient received neoadjuvant chemotherapy or radiotherapy. Adjuvant chemotherapy was administered to 167 patients (16.1%) at the clinicians' discretion based on tumor stage and overall health status. No patient received adjuvant radiotherapy.

### 2.2 Biomarker measurements

Plasma samples were collected after a preoperative overnight fast on the morning of the day of surgery. Specimen collection and measurement have been described in detail elsewhere.^28^ Briefly, blood was collected into Vacutainer CPT 8‐ml tubes containing 0.1 ml of molar sodium citrate (Becton Dickinson) and centrifuged at room temperature for 20 min at 1500*g*. The top layer corresponding to plasma was decanted using sterile transfer pipettes. The plasma was immediately frozen and stored at −80°C in polypropylene cryopreservation vials (NalgeNunc). For quantitative measurements of endoglin level, we used commercially available quantitative immunoassays (R&D Systems). Every sample was run in duplicate, and the mean was calculated for data analyses. The coefficient of variation was less than 10%.

### 2.3 Pathological review

All surgical specimens were processed according to standard pathological procedures. Genitourinary pathologists assigned tumor grades according to the 1973 WHO grading system. Pathological stage was reassigned according to the 2002 American Joint Committee on Cancer TNM staging system. The presence of concomitant carcinoma in situ (CIS) was defined as the presence of CIS in conjunction with another tumor other than CIS.^29^ Pelvic lymph nodes were examined grossly, and all lymphoid tissue was submitted for histological examination. Positive soft tissue surgical margin was defined as the presence of tumor at inked areas of soft tissue on the RC specimen.^30^ Urethral or ureteral margins were not considered soft tissue surgical margins. Lymphovascular invasion was defined as the unequivocal presence of tumor cells within an endothelium‐lined space without underlying muscular walls.^31^ Any nonorgan confined disease (NOCD) was defined as both ≥pT3 disease and lymph node metastasis.

### 2.4 Follow‐up

Clinical and radiological follow‐up was performed in accordance with institutional protocols and current guidelines. Routine follow‐up usually included physical examination, radiological imaging (CT of the thorax and abdomen), and urine cytology every 3 months for 2 years. Between the second and the fifth year, follow‐up was performed every 6 months. Afterward, in most cases, an annual follow‐up was performed. Tumor recurrence was defined as the occurrence of locoregional recurrence or distant metastasis on radiological imaging. Recurrence‐free survival (RFS) time was calculated from the date of RC to tumor recurrence or last follow‐up. Cause of death was abstracted from medical charts and/or from death certificates.^32^ Overall survival (OS) time was calculated from the date of RC to death or last follow‐up. Cancer‐specific survival (CSS) time was calculated from the date of RC to death from disease or last follow‐up.

### 2.5 Statistical analysis

Report of categorical variables included frequencies and proportions. Continuous variables were reported as medians and interquartile ranges (IQR). The median value of endoglin was calculated as 3.142 μg/ml, and this value was used as an independent endoglin cut‐off for analysis requiring a categorical variable such as Kaplan–Meier curve analysis and 5‐year survival comparisons. For all logistic and Cox regression models as well as for decision curve analysis (DCA), endoglin was considered as a continuous variable. With respect to preoperative plasma level of endoglin, group comparisons were performed using the Mann–Whitney *U*, Kruskal–Wallis, Wilcoxon rank‐sum, Pearson's *χ* ^2^, or Fisher's exact *t* tests, and subsequent significance testing, as appropriate.

Binominal logistic regression analysis was performed using preoperative available variables to evaluate the association of preoperative plasma level of endoglin with lymph node metastasis, ≥pT3 disease, or any NOCD. The risk of events was expressed as odds ratios (ORs) and 95% confidence intervals (95% CIs). The area under the curve (AUC) of receiver operating characteristics (ROC) curves was calculated to determine the predictive accuracy of multiple logistic regression models. DeLong's test was used to assess the additional predictive value of preoperative endoglin after adding it to a reference model by comparing the AUCs of the models.

Association between preoperative endoglin with RFS, CSS, and OS was assessed in univariable and multivariable Cox regression models. The risk of survival was expressed as hazard ratios (HRs) and 95% CI. Kaplan–Meier survival curves were used to depict the association between endoglin level and survival. The log‐rank test was used to determine the statistical difference between the endoglin (<3.142 and ≥3.142) groups with respect to recurrence or death. Two separate Cox regression models that featured either preoperative clinical variables or postoperative histopathological variables were created. Clinical and pathological tumor grade was excluded as a variable for all predictive models as virtually all RC patients had high‐grade UCB. The discriminative ability of the models before and after the inclusion of endoglin was tested and compared using Harrel's concordance indices (C‐index) to assess the additional prognostic value of endoglin. The additional clinical net‐benefit of endoglin was evaluated using DCA.^33^ All reported *p *values were two‐sided, and statistical significance was set at 0.05. All statistical analyses were performed using R Version 4.0.4.

## 3 RESULTS

### 3.1 Association of preoperative plasma endoglin level with clinicopathologic features

A total of 1036 patients were included in the analysis. The median age of the entire cohort was 67 years (IQR: 60–73). Patient characteristics are shown in Table 1. Median plasma levels of endoglin were significantly higher among patients with adverse pathologic features such as lymphovascular invasion (*p* < 0.001), lymph node metastasis (*p* < 0.001), contaminant CIS (*p* < 0.01), and advanced pathologic tumor stage (*p* < 0.001).

**Table 1:** Association of median preoperative plasma level of endoglin with clinicopathologic characteristics in 1036 patients treated with radical cystectomy for urothelial carcinoma of the bladder

Variable	Median plasma endoglin level, ng/ml (IQR)			Stratified by median preoperative plasma level of endoglin		
	Overall (*N* = 1036)	31.4 (20.0–39.2)	*p* value	Low ≥ 3.142 μg/ml (*N* = 515)	High < 3.142 μg/ml (*N* = 521)	*p* value
Age	67 (60, 73)	‐	‐	67 (61, 73)	66 (59, 72)	0.2
Gender			0.71			0.8
Male	814 (79%)	31.4 (19.6–39.1)		406 (79%)	408 (78%)	
Female	222 (21%)	31.5 (22.2–40.2)		109 (21%)	113 (22%)	
Blood transfusion			0.26			>0.9
No	768 (74%)	31.5 (19.7–39.1)		381 (74%)	387 (74%)	
Yes	268 (26%)	31.4 (20.4–39.9)		134 (26%)	134 (26%)	
Thrombocytosis			**0.047**			0.2
No	923 (89%)	31.2 (19.6–39.0)		465 (90.3%)	458 (88%)	
Yes	923 (89%)	33.5 (23.7–41.7)		50 (9.7%)	63 (12%)	
Hypoalbuminemia			0.06			0.2
No	891 (86%)	31.1 (19.9–39.0)		450 (87%)	441 (85%)	
Yes	145 (14%)	33.9 (21.8–40.0)		65 (13%)	80 (15%)	
Clinical tumor grade						>0.9
G2	6 (0.6%)			3 (0.6%)	3 (0.6%)	
G3	1022 (99%)	‐	‐	510 (99%)	512 (99%)	
Unknown	8			2	6	
Clinical tumor stage			***0.01***			**0.006**
cTa	23 (2.2%)	31.7 (13.3–39.6)		11 (2.1%)	12 (2.3%)	
cTis	105 (10%)	29.2 (19.4–39.1)		54 (11%)	51 (9.9%)	
cT1	336 (33%)	29.9 (20.2–37.9)		184 (36%)	152 (29%)	
cT2	498 (48%)	32.7 (19.5–39.7)		233 (45%)	265 (51%)	
cT3	38 (3.7%)	37.3 (30.2–43.4)		11 (2.1%)	27 (5.2%)	
cT4	29 (2.8%)	23.5 (21.0–40.1)		20 (3.9%)	9 (1.7%)	
Unknown	7	40.4 (27.1–47.7)		2	5	
Pathological tumor grade			0.05			0.2
G1	62 (6.0%)	35.3 (24.1–41.5)		25 (4.9%)	37 (7.1%)	
G2	11 (1.1%)	19.4 (13.9–38.5)		7 (1.4%)	4 (0.8%)	
G3	963 (93%)	31.3 (19.6–39.1)		483 (94%)	480 (92%)	
Pathological tumor stage			**<0.001**			**<0.001**
pT0	62 (6.0%)	35.3 (24.1–41.5)		25 (4.9%)	37 (7.1%)	
pTa	22 (2.1%)	43.1 (14.1–48.6)		9 (1.7%)	13 (2.5%)	
pTis	131 (13%)	24.6 (11.1–36.9)		72 (14%)	59 (11%)	
pT1	162 (16%)	27.1 (12.7–37.4)		96 (19%)	66 (13%)	
pT2	248 (24%)	27.3 (17.7–36.3)		164 (32%)	84 (16%)	
pT3	281 (27%)	33.3 (26.0–39.1)		103 (20%)	178 (34%)	
pT4	130 (13%)	38.6 (23.2–46.1)		46 (8.9%)	84 (16%)	
Positive soft tissue surgical margins			**<0.001**			**0.001**
No	941 (91%)	30.8 (19.3–38.8)		483 (93.8%)	458 (88%)	
Yes	95 (9.2%)	35.6 (25.7–43.7)		32 (6.2%)	63 (12%)	
Lymphovascular invasion			***<0.001***			**0.004**
No	741 (72%)	30.1 (18.0–39.0)		389 (76%)	352 (58%)	
Yes	295 (28%)	33.3 (23.7–40.4)		126 (24%)	169 (32%)	
Concomitant CIS			**<0.01**			0.5
No	464 (45%)	32.0 (22.2–39.8)		225 (44%)	239 (46%)	
Yes	572 (55%)	31.1 (18.0–39.1)		290 (56%)	282 (54%)	
Lymph node involvement			**<0.001**			**<0.001**
No	773 (75%)	27.3 (16.6–38.5)		439 (85%)	334 (64%)	
Yes	263 (25%)	34.9 (30.2–41.6)		76 (15%)	187 (36%)	
Adjuvant chemotherapy			**<0.001**			**0.01**
No	869 (84%)	30.5 (18.9–39.1)		447 (87%)	422 (81%)	
Yes	167 (16%)	33.3 (26.5–40.6)		68 (13%)	99 (19%)	

*Note*: Median (IQR); *n* (%). Bold *p* values are statistically significant.

On multivariable logistic regression modeling, elevated preoperative plasma levels of endoglin were significantly associated with an increased risk of lymph node metastasis, ≥pT3 disease, and any NOCD (all *p* < 0.001) (Table 2). ROC curve analyses showed that the addition of preoperative plasma levels of endoglin to a reference model comprising age, sex, and clinical tumor stage improved the discriminatory ability for the prediction of lymph node metastasis (10%, *p* < 0.001), ≥pT3 disease (5%, *p* < 0.001), and any NOCD (5.8%, *p* < 0.001).

**Table 2:** Multivariable logistic regression models for the prediction of lymph node metastasis, ≥pT3 disease, and any nonorgan confined disease in 1029 patients treated with radical cystectomy for urothelial carcinoma of the bladder

Variable	Lymph node involvement			≥pT3 disease			Any nonorgan confined disease		
	OR	95% CI	*p* value	OR	95% CI	*p* value	OR	95% CI	*p* value
Endoglin	1.72	1.52, 1.97	**<0.001**	1.53	1.37, 1.72	**<0.001**	1.61	1.44, 1.81	**<0.001**
Age	1.00	0.98, 1.01	0.9	1.03	1.01, 1.04	**<0.001**	1.02	1.01, 1.04	**0.001**
Gender (female)	1.40	0.98, 1.99	0.06	1.02	0.73, 1.42	0.9	1.14	0.82, 1.59	0.4
Clinical tumor stage									
cTa/cTis/cT1	Ref	Ref	Ref	Ref	Ref	Ref	Ref	Ref	Ref
cT2	2.38	1.72, 3.30	**<0.001**	2.64	1.99, 3.51	**<0.001**	2.96	2.25, 3.91	**<0.001**
≥cT3	3.13	1.74, 5.55	**<0.001**	8.83	4.89, 16.7	**<0.001**	8.25	4.44, 16.2	**<0.001**
AUC with endoglin	0.733			0.725			0.734		
AUC without endoglin	0.629			0.675			0.676		
DeLong's test *p* value	**<0.001**			**<0.001**			**<0.001**		

*Note*: Bold *p* values are statistically significant.

Abbreviations: AUC, area under the curve; CI, confidence interval; OR, odds ratio. CI, confidence interval.

**Figure 1:**
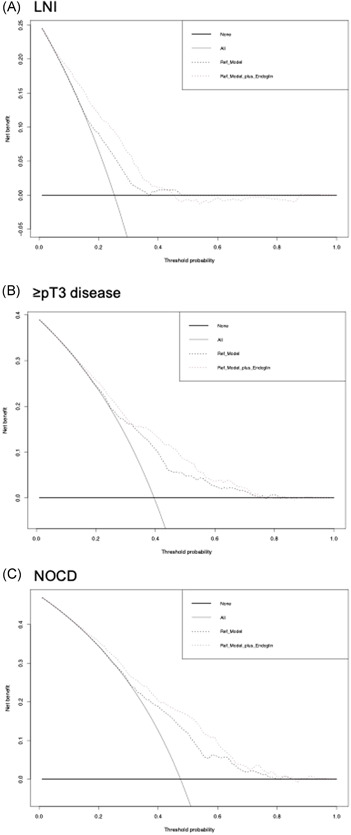
Decision curve analyses (DCA) for the evaluation of the clinical net‐benefit using the log models for the prediction of (A) lymph node metastasis, (B) ≥ pT3 disease, and (C) any nonorgan confined disease

On DCA for prediction of lymph node metastasis, the addition of preoperative endoglin plasma levels to the preoperative standard model resulted in the improved clinical net‐benefit between a threshold probability of 30%–60% (Figure 1A); 67.8% of patients would benefit from the endoglin model for prediction of lymph node metastasis. DCA for prediction of both ≥pT3 disease and any NOCD revealed that the addition of preoperative plasma level of endoglin to the preoperative standard model resulted only in a slight improvement of the clinical net‐benefit (Figure 1B,C). Only 28.1% of patients would benefit from the endoglin model for prediction of ≥pT3 disease, and 27.4% of patients would benefit from the endoglin model for prediction of any NOCD.

### 3.2 Association of survival outcomes within a preoperative model

**Figure 2:**
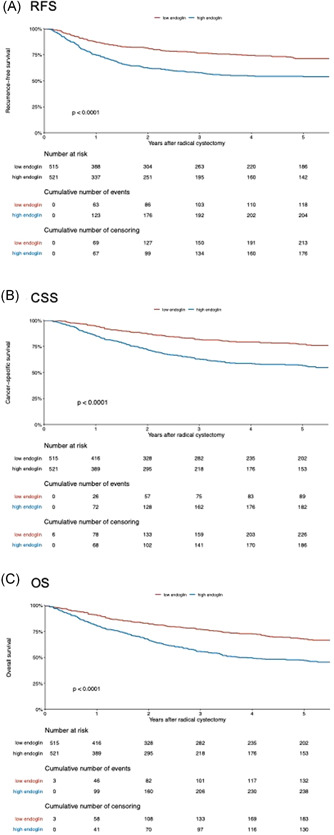
Kaplan–Meier analysis for (A) recurrence‐free survival (RFS), (B) cancer‐specific survival (CSS), and (C) overall survival (OS) in 1036 patients treated with radical cystectomy for urothelial carcinoma of the bladder, stratified according to preoperative plasma level of endoglin at a cut‐off of 3.142 [Color figure can be viewed at wileyonlinelibrary.com]

Median follow‐up of patients alive was 37 months (IQR: 14.5–108.5). Overall, the 5‐year estimates for RFS, CSS, and OS were 62.5% (95% CI: 59.2%–66%), 66% (95% CI: 63.3%−70%), and 57% (95% CI: 53.6%–60.5%), respectively. In patients with low versus high median levels of preoperative endoglin, the 5‐year RFS, CSS, and OS were 71% (95% CI: 67%–76%) versus 53.8% (95% CI: 49%–59%), 77% (95% CI: 72.8%–81.5%) versus 56.5% (95% CI: 51.9%–61.7%), and 67.6% (95% CI: 63%–72.5%) versus 47% (95% CI: 42.4%–52%), respectively. Higher preoperative plasma level of endoglin was associated with worse RFS (HR: 1.85, 95% CI: 1.49–2.31, *p* < 0.001), CSS (HR: 2.02, 95% CI: 1.60–2.55, *p* < 0.001), and OS (HR 1.63, 95% CI: 1.38–1.92, *p* < 0.001) (Figure 2).

**Figure 3:**
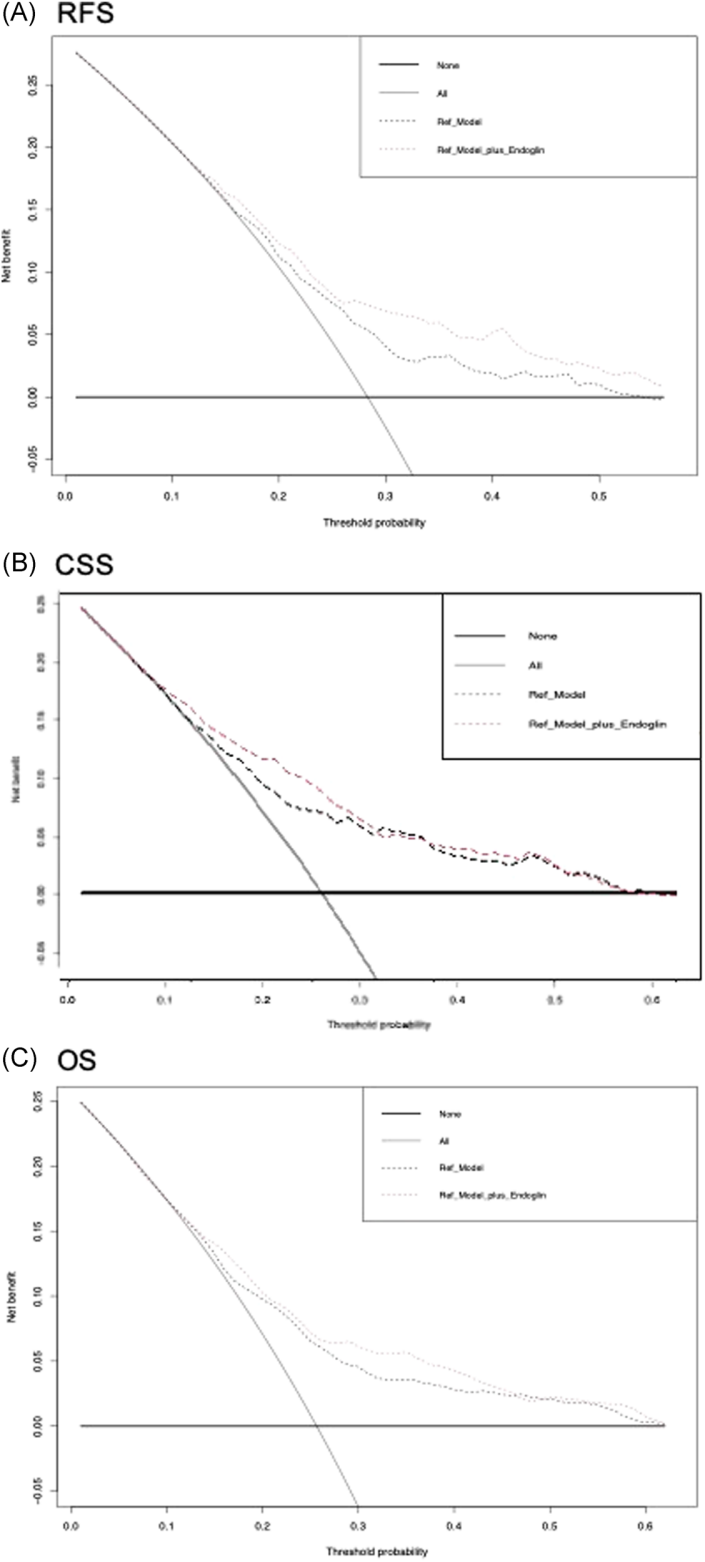
Decision curve analyses (DCA) for the evaluation of the clinical net‐benefit using the preoperative Cox models for the prediction of (A) recurrence‐free survival (RFS), (B) cancer‐specific survival (CSS), and (C) overall survival (OS) [Color figure can be viewed at wileyonlinelibrary.com]

In a multivariable Cox regression model that included established available preoperative variables (age, sex, and clinical tumor stage), a higher preoperative plasma level of endoglin was associated with worse RFS, CSS, and OS (all *p* < 0.001) (Table 3). The addition of preoperative plasma levels of endoglin slightly improved the C‐indices of the same model for prediction of early RFS (4%), CSS (4.6%), and OS (2.7%). On DCA, the addition of preoperative endoglin plasma level to the same Cox model slightly improved the clinical net‐benefit of the model for early prediction of RFS with a probability between 30% and 50%, while there was no improvement for the prediction of CSS or OS (Figure 3).

**Table 3:** Separate pre‐ and postoperative multivariable Cox regression models for the prediction of recurrence‐free survival, cancer‐specific survival, and overall survival in 1036 patients treated with radical cystectomy for urothelial carcinoma of the bladder

Variable	Recurrence‐free survival			Cancer‐specific survival			Overall survival		
	HR	95% CI	*p* value	HR	95% CI	*p* value	HR	95% CI	*p* value
**Preoperative model**									
Endoglin	1.28	1.18, 1.39	**<0.001**	1.33	1.22, 1.45	**<0.001**	1.21	1.14, 1.29	**<0.001**
Age	1.02	1.01, 1.03	**0.002**	1.02	1.01, 1.04	**<0.001**	1.05	1.04, 1.06	**<0.001**
Gender (female)	1.48	1.15, 1.89	**0.002**	1.59	1.23, 2.05	**<0.001**	1.30	1.07, 1.58	**0.009**
Clinical tumor stage									
cTa/cTis/cT1	Ref	Ref	Ref	Ref	Ref	Ref	Ref	Ref	Ref
cT2	1.66	1.32, 2.10	**<0.001**	1.77	1.38, 2.27	**<0.001**	1.59	1.33, 1.90	**<0.001**
≥cT3	1.97	1.31, 2.96	**0.001**	2.23	1.47, 3.40	**<0.001**	1.88	1.37, 2.59	**<0.001**
C‐index with endoglin	0.645			0.678			0.663		
C‐index without endoglin	0.605			0.632			0.636		
**Postoperative model**									
Endoglin	1.09	0.99, 1.20	0.06	1.13	1.02, 1.24	**0.02**	1.11	1.04, 1.19	**0.003**
Age	1.01	1.00, 1.02	0.09	1.02	1.00, 1.03	**0.01**	1.04	1.03, 1.05	**<0.001**
Gender (female)	1.53	1.19, 1.96	**<0.001**	1.59	1.23, 2.06	**<0.001**	1.35	1.11, 1.64	**0.003**
Pathological stage									
pT0/pTa/pTis/pT1	Ref	Ref	Ref	Ref	Ref	Ref	Ref	Ref	Ref
pT2	1.51	1.04, 2.18	**0.03**	1.47	0.99, 2.18	0.05	1.39	1.09, 1.77	**0.01**
≥pT3	3.11	2.19, 4.40	**<0.001**	2.99	2.06, 4.32	**<0.001**	2.43	1.90, 3.11	**<0.001**
Positive soft tissue surgical margins	1.35	0.99, 1.85	0.06	1.42	1.02, 1.96	**0.04**	1.05	0.79, 1.39	0.7
Lymphovascular invasion	1.43	1.11, 1.83	**0.005**	1.58	1.22, 2.05	**<0.001**	1.24	1.01, 1.51	**0.04**
Concomitant CIS	1.03	0.83, 1.29	0.8	0.94	0.74, 1.19	0.6	1.01	0.85, 1.20	0.9
Lymph node involvement	2.36	1.81, 3.06	**<0.001**	2.41	1.84, 3.16	**<0.001**	1.97	1.59, 2.43	**<0.001**
Adjuvant chemotherapy	0.91	0.70, 1.20	0.5	0.97	0.73, 1.28	0.8	0.85	0.68, 1.08	0.2
C‐index with endoglin	0.751			0.777			0.734		
C‐index without endoglin	0.754			0.778			0.733		

*Note*: Bold *p* values are statistically significant.

Abbreviations: CI, confidence interval; CIS, carcinoma in situ; HR, hazard ratio.

In the subgroup analyses of 498 patients with the cT2 clinical‐stage, an elevated preoperative plasma level of endoglin was independently associated with worse RFS, CSS, and OS (all *p* < 0.001) (Table S1). The addition of preoperative plasma levels of endoglin improved the C‐indices of the same model for early prediction of RFS (8.1%), CSS (8.2%), and OS (4%). In the subgroup analyses of 336 patients with cT1 clinical stage, the preoperative plasma levels of endoglin failed to have an association with RFS, CSS, or OS (all *p* > 0.05) (Table S2).

### 3.3 Association of survival outcomes within a postoperative model

**Figure 4:**
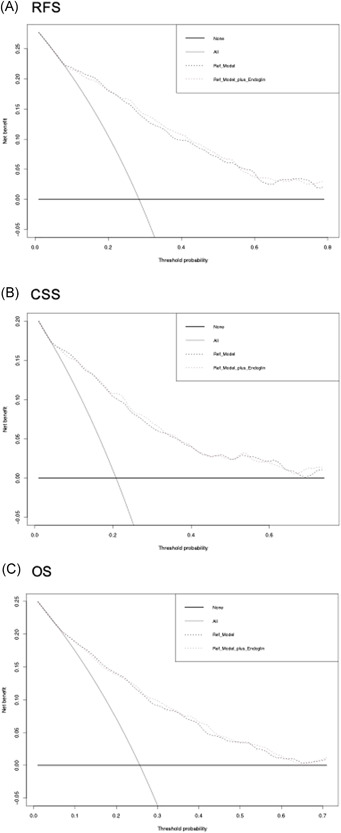
Decision curve analyses (DCA) for the evaluation of the clinical net‐benefit using the postoperative Cox models for the prediction of (A) recurrence‐free survival (RFS), (B) cancer‐specific survival (CSS), and (C) overall survival (OS)

In a multivariable Cox regression model that included established postoperative variables, elevated preoperative plasma level of endoglin remained independently associated with worse CSS and OS (*p* = 0.02 and *p* = 0.003, respectively), but not anymore with RFS (Table 3). The addition of preoperative plasma level of endoglin from a base prognostic model that included established postoperative variables did not result in an increase of the C‐indices for prediction of RFS, CSS, or OS. On DCA, the addition of preoperative plasma level of endoglin did not improve the clinical net‐benefit of the models for the prediction of either RFS, CSS, or OS (Figure 4).

In the subgroup analyses of 190 patients with pT2N0 stage, an elevated preoperative plasma level of endoglin was independently associated with worse RFS and CSS (all *p* < 0.001) (Table S3). The exclusion of preoperative plasma level of endoglin from a base prognostic model that included established postoperative variables resulted in a decrease of the C‐indices for prediction of both RFS (12.5%) and CSS (11.4%). Similarly, in the subgroup of patients with ≥pT3 stage, the elevated preoperative plasma levels of endoglin were independently associated with worse RFS, CSS, and OS (all *p* < 0.001) (Table S4); differential increases in C‐indices were 5.5% for RFS, 4.1% for CSS, and 2.4% for OS.

## 4 DISCUSSION

To our best knowledge, this is the first study aimed to determine the predictive and prognostic value of preoperative plasma level of endoglin in patients with urothelial carcinoma. The present large‐scale study led to several important findings.

According to our results, preoperative plasma endoglin level was a strong predictor of lymph node metastasis, ≥pT3 disease, and any NOCD in patients treated with RC for nonmetastatic UCB. The addition of endoglin to established preoperative variables improved the ability to predict lymph node metastasis by a statistically and clinically significant margin, while for ≥pT3 disease and NOCD prediction, the margin was minimal. This is in agreement with previous studies reporting preoperative plasma endoglin to improve the accuracy for the prediction of pelvic lymph node metastasis in patients treated with radical prostatectomy for clinically localized prostate cancer.^25^, ^27^ In contrast, Gomceli et al. failed to find an additive predictive value to endoglin levels in patients with gastric or colorectal carcinoma.^34^, ^35^ Conversely, in accordance with our findings, Daly et al. found endoglin levels to increase in the transition from node‐positive disease to disseminated disease in patients with lung adenocarcinoma.^24^ Similarly, several independent research groups reported an association of plasma endoglin with distant metastasis in patients with both colorectal and breast cancers.^22^, ^36^ Findings from these studies suggested that plasma endoglin levels are associated with the metastatic process and may be useful in the identification of early metastases. However, it seems that the prediction probability of endoglin highly depends on tumor biology and other factors. Our promising results on the prediction of biologically and clinically aggressive UCB should be, therefore, externally validated in other cohorts. Although an association with pathologic features is of importance as it allows for tailored pre‐ and intraoperative strategies, prognostication of survival outcomes would allow for personalized decision‐making regarding perioperative treatment.

We found an independent association of elevated preoperative endoglin with worse survival outcomes (RFS, CSS, and OS) in multivariable Cox regression models that adjusted for the effects of both established preoperative and postoperative variables. Additionally, we found that preoperative endoglin improved the survival prediction of the preoperative model in the subgroup of cT2 patients as well as the postoperative model in the subgroup of patients with pT2N0 and ≥pT3 UCB. Plasma levels of endoglin reflect micrometastases that are hypothesized to be higher in more extensive or aggressive tumors. This makes endoglin a promising tool in the identification of cT2 UCB patients who are most likely to benefit from neoadjuvant chemotherapy, pT2N0 patients who are most likely to benefit from adjuvant systemic therapy, and ≥pT3 patients in whom adjuvant systemic therapy could be safely avoided.

Nevertheless, conventional multivariable analyses and the change in C‐index or AUC that quantify the ability of the model to discriminate between patients with and those without the outcome of interest are not sufficient to demonstrate that a biomarker provides a clinical benefit.^19^ To explore the net benefit of adding of endoglin to the standard models, we performed DCA, a method that combines simplicity with efficient computations.^19^ According to DCA, in our study, the addition of endoglin improved the clinical net benefit of the standard model for the prediction of lymph node metastasis; indeed, 67.8% of patients would benefit from the novel model featuring endoglin. For the prediction of ≥pT3 disease or any NOCD, it only marginally improved the net benefit by what is estimated to be a clinically nonsignificant margin. In contrast, endoglin did not improve the net clinical benefit in the pre‐ and postoperative setting for survival outcomes. Hence, it seems that clinicians can use preoperative blood‐based endoglin for a more accurate estimation of the probability of lymph node metastasis in patients with nonmetastatic UCB thereby allowing counseling of patients regarding intensified perioperative systemic therapy and extent of lymphadenectomy.

It is also important to consider that combining preoperative plasma endoglin with other blood‐based biomarkers is more likely to capture a higher predictive value than any single biomarkers.^9^, ^19^, ^37^, ^38^ Additionally, its combination with tissue expression of endoglin, as a marker of angiogenesis, in a specimen from transurethral resection of bladder tumor might help in preoperative patient counseling, especially in patients with papillary noninvasive bladder cancer as a tumor with well‐developed branching fibrovascular core. Urothelium endoglin antibodies have been shown to recognize small‐caliber vessels that are associated with angiogenesis in bladder cancer that can help identify high‐risk patients who could benefit from antiangiogenic therapeutic regimens.^39^ Moreover, a novel targeted therapy with monoclonal antibodies that binds endoglin (CD105) is under investigation in patients with advanced solid tumors.^40^ Endoglin might be a useful marker for tumor angiogenesis detection in studies testing novel targeted therapy in combination with chemotherapy and VEGF inhibitors as well as a single agent.

The main strength of the present large‐scale study is that, to our knowledge, this is the first to evaluate the prognostic value of preoperative plasma level of endoglin in patients treated with RC for nonmetastatic UCB. Nevertheless, our study is not devoid of limitations. The main limitation of the study was its retrospective and multicenter design, which may result in a lack of pathologic and surgical approaches that could confound the results. Another limitation of our study is the fact that confounding conditions, such as undiagnosed infectious diseases or unknown drug interaction, could potentially have affected plasma levels of endoglin. However, this would have weakened an existing potential association. Further, data on therapies before RC, such as intravesical bacillus calmette‐guérin instillations, which might also alter levels of endoglin, were, unfortunately, unavailable. The strength of this cohort is its homogeneity in treatment allocation that demonstrates the full biologic effect of endoglin. However, at the same time, its weakness is that it does not reflect current treatment standards. Due to the time of recruitment of this study, no patient received neoadjuvant chemotherapy. Ideally, the contemporary predictive value of plasma endoglin should be assessed in the neoadjuvant setting. Additionally, endoglin was assessed preoperatively at a single time point. Hence, endoglin variability over time and in response to treatment has not been tested. However, it has been reported that endoglin level changes in lung cancer patients after surgical treatment.^41^ Future studies should assess this theory in the context of urothelial carcinoma. Another limitation is the short follow‐up period with a median of 37 months. However, it was shown that over two‐thirds of patients experience disease recurrence within 12 months after RC and ≥90% within 24 months.^42^ Despite all these limitations, we presented the largest series investigating the association of preoperative endoglin with oncologic outcomes in patients treated with RC for nonmetastatic UCB. Further well‐designed studies should be conducted to validate our promising results.

## 5 CONCLUSION

Preoperative plasma endoglin holds potential in identifying UCB patients who may benefit from intensified therapy in addition to RC due to its association with features of the biologically and clinically aggressive disease as well as poor survival outcomes. In particular, with respect to the prediction of lymph node metastasis, preoperative endoglin offers a high discriminatory power, which warrants inclusion into future predictive models.

## CONFLICT OF INTERESTS

The authors declare that there are no conflict of interests.

## AUTHOR CONTRIBUTIONS

All authors made substantial contributions to the conception and design of the study, acquisition of data, analysis and interpretation of data, drafting the article and revising it critically for important intellectual content as well as final approval of the version to be submitted.

## Supporting information

Supporting information.

## Data Availability

The data that support the findings of this study are available from the corresponding author upon reasonable request.
