# Supplementary material for: Preoperative plasma level of endoglin as a predictor for disease outcomes after radical cystectomy for nonmetastatic urothelial carcinoma of the bladder
Source: Mol Carcinog. 2021 Sep 29;61(1):5–18. doi: 10.1002/mc.23355 (PMC9293216; doi:10.1002/mc.23355)
Supplement: Supplementary file 1 — Supporting information. [file MC-61-5-s001.docx]

**Table S1. Preoperative multivariable Cox regression models for the prediction of recurrence-free survival, cancer-specific survival, and overall survival in 498 patients with cT2 clinical stage treated with radical cystectomy for urothelial carcinoma of the bladder.**

| Variable | Recurrence-free survival | | | Cancer-specific survival | | | Overall survival | | |
| --- | --- | --- | --- | --- | --- | --- | --- | --- | --- |
|  | HR | 95% CI | p-value | HR | 95% CI | p-value | HR | 95% CI | p-value |
| Endoglin | 1.03 | 1.02, 1.05 | **<0.001** | 1.04 | 1.02, 1.05 | **<0.001** | 1.03 | 1.02, 1.03 | **<0.001** |
| Age | 1.01 | 1.00, 1.03 | 0.06 | 1.02 | 1.00, 1.03 | **0.03** | 1.05 | 1.03, 1.06 | **<0.001** |
| Gender (female) | 1.45 | 1.04, 2.03 | **0.03** | 1.59 | 1.13, 2.24 | **0.01** | 1.39 | 1.07, 1.81 | **0.01** |
| C-index with endoglin | 0.643 | | | 0.659 | | | 0.649 | | |
| C-index without endoglin | 0.562 | | | 0.577 | | | 0.609 | | |

**Table S2. Preoperative multivariable Cox regression models for the prediction of recurrence-free survival, cancer-specific survival, and overall survival in 336 patients with cT1 clinical stage treated with radical cystectomy for urothelial carcinoma of the bladder.**

| Variable | Recurrence-free survival | | | Cancer-specific survival | | | Overall survival | | |
| --- | --- | --- | --- | --- | --- | --- | --- | --- | --- |
|  | HR | 95% CI | p-value | HR | 95% CI | p-value | HR | 95% CI | p-value |
| Endoglin | 1.01 | 0.99, 1.03 | 0.2 | 1.01 | 0.99, 1.03 | 0.2 | 1.01 | 1.00, 1.02 | 0.2 |
| Age | 1.03 | 1.00, 1.05 | **0.02** | 1.03 | 1.01, 1.06 | **0.01** | 1.05 | 1.03, 1.08 | **<0.001** |
| Gender (female) | 1.31 | 0.80, 2.16 | 0.3 | 1.48 | 0.89, 2.48 | 0.13 | 1.08 | 0.73, 1.60 | 0.7 |
| C-index with endoglin | 0.588 | | | 0.612 | | | 0.619 | | |
| C-index without endoglin | 0.571 | | | 0.595 | | | 0.611 | | |

**Table S3. Postoperative multivariable Cox regression models for the prediction of recurrence-free survival, cancer-specific survival, and overall survival in 190 patients with pT2N0 stage treated with radical cystectomy for urothelial carcinoma of the bladder.**

| Variable | Recurrence-free survival | | | Cancer-specific survival | | | Overall survival | | |
| --- | --- | --- | --- | --- | --- | --- | --- | --- | --- |
|  | HR | 95% CI | p-value | HR | 95% CI | p-value | HR | 95% CI | p-value |
| Endoglin | 0.91 | 0.87, 0.95 | **<0.001** | 0.91 | 0.87, 0.95 | **<0.001** | 1.00 | 0.99, 1.02 | 0.9 |
| Age | 1.02 | 0.98, 1.06 | 0.3 | 1.03 | 0.99, 1.08 | 0.14 | 1.08 | 1.06, 1.11 | **<0.001** |
| Gender (female) | 1.67 | 0.74, 3.77 | 0.2 | 2.01 | 0.85, 4.76 | 0.11 | 1.59 | 0.96, 2.63 | 0.07 |
| Positive soft tissue surgical margins | 3.28 | 1.62, 6.66 | **0.001** | 4.54 | 2.06, 10.0 | **<0.001** | 2.37 | 1.47, 3.81 | **<0.001** |
| Lymphovascular invasion | 3.28 | 1.62, 6.66 | **0.001** | 4.54 | 2.06, 10.0 | **<0.001** | 2.37 | 1.47, 3.81 | **<0.001** |
| Concomitant CIS | 1.38 | 0.66, 2.90 | 0.4 | 1.19 | 0.53, 2.68 | 0.7 | 1.00 | 0.64, 1.57 | >0.9 |
| Adjuvant chemotherapy | 0.98 | 0.22, 4.42 | >0.9 | 1.13 | 0.25, 5.13 | 0.9 | 0.48 | 0.19, 1.25 | 0.13 |
| C-index with endoglin | 0.815 | | | 0.832 | | | 0.692 | | |
| C-index without endoglin | 0.690 | | | 0.718 | | | 0.692 | | |

**Table S4. Postoperative multivariable Cox regression models for the prediction of recurrence-free survival, cancer-specific survival, and overall survival in 411 patients with ≥pT3 stage treated with radical cystectomy for urothelial carcinoma of the bladder.**

| Variable | Recurrence-free survival | | | Cancer-specific survival | | | Overall survival | | |
| --- | --- | --- | --- | --- | --- | --- | --- | --- | --- |
|  | HR | 95% CI | p-value | HR | 95% CI | p-value | HR | 95% CI | p-value |
| Endoglin | 1.03 | 1.02, 1.04 | **<0.001** | 1.03 | 1.02, 1.04 | **<0.001** | 1.02 | 1.02, 1.03 | **<0.001** |
| Age | 1.00 | 0.99, 1.02 | 0.8 | 1.01 | 0.99, 1.02 | 0.5 | 1.02 | 1.01, 1.03 | **0.005** |
| Gender (female) | 1.47 | 1.08, 2.00 | **0.01** | 1.48 | 1.07, 2.04 | **0.02** | 1.36 | 1.03, 1.80 | **0.03** |
| Positive soft tissue surgical margins | 1.46 | 1.05, 2.04 | **0.03** | 1.47 | 1.04, 2.08 | **0.03** | 1.17 | 0.86, 1.59 | 0.3 |
| Lymphovascular invasion | 1.55 | 1.17, 2.07 | **0.002** | 1.77 | 1.31, 2.39 | **<0.001** | 1.44 | 1.13, 1.85 | **0.003** |
| Concomitant CIS | 1.23 | 0.93, 1.62 | 0.14 | 1.17 | 0.87, 1.56 | 0.3 | 1.18 | 0.93, 1.51 | 0.2 |
| Adjuvant chemotherapy | 0.99 | 0.73, 1.35 | >0.9 | 1.03 | 0.74, 1.42 | 0.9 | 0.98 | 0.75, 1.29 | >0.9 |
| C-index with endoglin | 0.656 | | | 0.680 | | | 0.655 | | |
| C-index without endoglin | 0.611 | | | 0.639 | | | 0.631 | | |
